# Development of a four autophagy-related gene signature for active tuberculosis diagnosis

**DOI:** 10.3389/fcimb.2025.1600348

**Published:** 2025-05-23

**Authors:** Baoyan Ren, Feng Jia, Qixun Fang, Jingping Xu, Kangfeng Lin, Renhui Huang, Zhenqiong Liu, Xingan Xing

**Affiliations:** ^1^ Yaneng Bioscience Co. Ltd., Shenzhen, Guangdong, China; ^2^ Department of Clinical Laboratory, Jiangxi Provincial Chest Hospital, Nanchang, Jiangxi, China; ^3^ School of Chemistry and Chemical Engineering, South China University of Technology, Guangzhou, Guangdong, China

**Keywords:** tuberculosis, whole blood, signature, diagnosis, autophagy

## Abstract

**Background:**

Tuberculosis (TB) diagnostics urgently require non-sputum biomarkers to address the limitations of conventional methods in distinguishing active TB (ATB) from latent infection (LTBI), healthy controls (HCs), and TB-mimicking diseases (ODs, other diseases).

**Methods:**

Transcriptomic data from GSE83456 and GSE152532 were combined to form the selection dataset. Marker genes were identified from differentially expressed autophagy-related genes using a Random Forest classifier. The optimal gene signature was selected based on optimal performance through a linear Support Vector Machine (SVM) classifier with cross-validation. The signature was subsequently evaluated in six independent evaluation datasets and validated using whole blood samples collected from 70 participants.

**Results:**

We identified a novel four-gene autophagy-related signature (*CASP1*, *FAS*, *TRIM5*, *C5*) in the selection dataset. This signature demonstrated robust diagnostic accuracy across multiple evaluation datasets: Area Under the Curve (AUC) 0.83–0.98 for ATB vs. LTBI and 0.82–0.94 for ATB vs. HCs. Crucially, it maintained high specificity (AUC 0.89–0.90) against ODs. RT-qPCR validation in whole blood samples confirmed elevated expression in ATB, while an SVM model achieved promising differentiation (AUC 0.86 for ATB vs. LTBI and AUC 0.99 for ATB vs. HCs).

**Conclusions:**

Our findings yielded a four-gene signature in whole blood that is robustly diagnostic for ATB, validated across multiple evaluation datasets and clinical samples. The autophagy-driven specificity and PCR-compatible design of this signature offer a blood-based, cost-effective strategy to enhance TB detection, addressing WHO-aligned diagnostic needs.

## Introduction

Tuberculosis (TB), caused by *Mycobacterium tuberculosis* (Mtb), remains a leading global cause of morbidity and mortality, with an estimated 10.8 million new cases and 1.25 million deaths in 2023 (World Health Organization, 2024). Despite the intricate interplay between Mtb replication and host immune defenses, infected individuals may remain asymptomatic, or progress to LTBI or ATB (Chandra et al., 2022). ATB poses a particularly urgent challenge due to its high transmissibility and the limitations of current diagnostic tools. Conventional sputum-based TB diagnosis methods, such as sputum smear microscopy and culture, exhibit low sensitivity and prolonged turnaround times, while molecular assays like Xpert MTB/RIF, though rapid, depend on sputum quality (Datta et al., 2017). Furthermore, existing immunodiagnostic tests, such as tuberculin skin tests (TST) and interferon-gamma release assays (IGRAs), cannot distinguish ATB from LTBI or predict progression to disease (Carranza et al., 2020). These gaps underscore the urgent need for non-sputum biomarkers that reflect host-pathogen dynamics and enable early, accurate diagnosis.

Recent advances in transcriptomics have identified blood-based gene signatures as promising solutions. Early research identified a 393-gene signature in ATB through blood transcriptomic profiling (Berry et al., 2010), which highlights the potential of blood-based biomarkers. Subsequent studies identified a large number of reduced gene number signatures for the diagnosis of ATB. For instance, Sweeney3 (GBP5, DUSP3, and KLF2) discriminated ATB from LTBI, healthy controls (HCs) and other diseases (OD) with promising sensitivity and specificity across diverse cohorts (Sweeney et al., 2016; Sutherland et al., 2022). Similarly, RISK6, a 6-gene signature, demonstrated robust performance for distinguishing ATB in multi-country evaluations, meeting WHO target product profiles for non-sputum triage tests (Penn-Nicholson et al., 2020). However, transcriptomic signatures face critical limitations. Their diagnostic accuracy diminishes in subclinical TB and HIV-coinfected populations, while cross-reactivity with viral infections and non-TB inflammatory conditions compromises specificity (Turner et al., 2020; Mendelsohn et al., 2022). Validation studies in real-world cohorts have demonstrated suboptimal diagnostic accuracy of these signatures in clinical practice (Hoang et al., 2021). These challenges underscore the need for identifying novel transcriptomic signatures and optimizing gene combinations to enhance accuracy and clinical utility in TB diagnosis.

Autophagy, a lysosomal degradation process essential for cellular homeostasis, plays dual roles in clearing damaged organelles and combating microbial invaders. During Mtb infection, autophagy acts as a critical host defense mechanism by restricting intracellular bacterial survival through phagolysosomal fusion and by delivering pathogen-derived components to immune receptors (Kim et al., 2019). Experimental models, including human macrophages and murine systems, demonstrate that genetic ablation of autophagy-related genes (ARGs) such as ATG5 and ATG7 exacerbates Mtb replication and promotes necroptotic cell death, rendering hosts more susceptible to infection (Gutierrez et al., 2004; Kimmey et al., 2015; Wang et al., 2023). Strategies to enhance autophagic flux have shown promise in restricting the survival of Mtb (Pahari et al., 2020). Conversely, Mtb subverts autophagy through virulence effectors (e.g., ESX-1, PDIMs), blocking autophagosome maturation to create a permissive niche for intracellular replication and immune evasion (Koster et al., 2017; Mittal et al., 2024). This tug-of-war underscores autophagy’s centrality in Mtb-host dynamics, with emerging evidence suggesting that autophagy-related biomarkers could enhance tuberculosis diagnostics by distinguishing latent from active infection. Targeting this pathway may thus offer clinical diagnostic benefits.

Here, we hypothesize that a combinatorial ARG signature in peripheral blood can robustly discriminate ATB from LTBI, HC, and OD. To address the critical unmet need for non-sputum biomarkers, we integrated transcriptomic profiling of ARGs with machine learning-driven feature selection, identifying a novel four-gene signature (*CASP1*, *FAS*, *TRIM5*, and *C5*) through differential expression analysis and random forest validation. This signature demonstrated high diagnostic specificity for ATB across independent cohorts and standardized RT-qPCR assays. By bridging autophagy biology and clinical diagnostics, our work could deliver a blood-based signature as a cost-effective and translatable tool for ATB detection, advancing precision in global TB management.

## Materials and methods

### Data acquisition

Public transcriptomic datasets were retrieved from the Gene Expression Omnibus (GEO; https://www.ncbi.nlm.nih.gov/geo/), using the search terms: “tuberculosis blood gene expression”, “tuberculosis blood microarray”, and “tuberculosis blood RNAseq”. Datasets were selected based on the following criteria: adult whole-blood samples, genome-wide profiling platforms (microarray or RNA sequencing), and exclusion of cohorts with small sample sizes (<10 per group) or incomplete clinical annotations.

**Figure 1:**
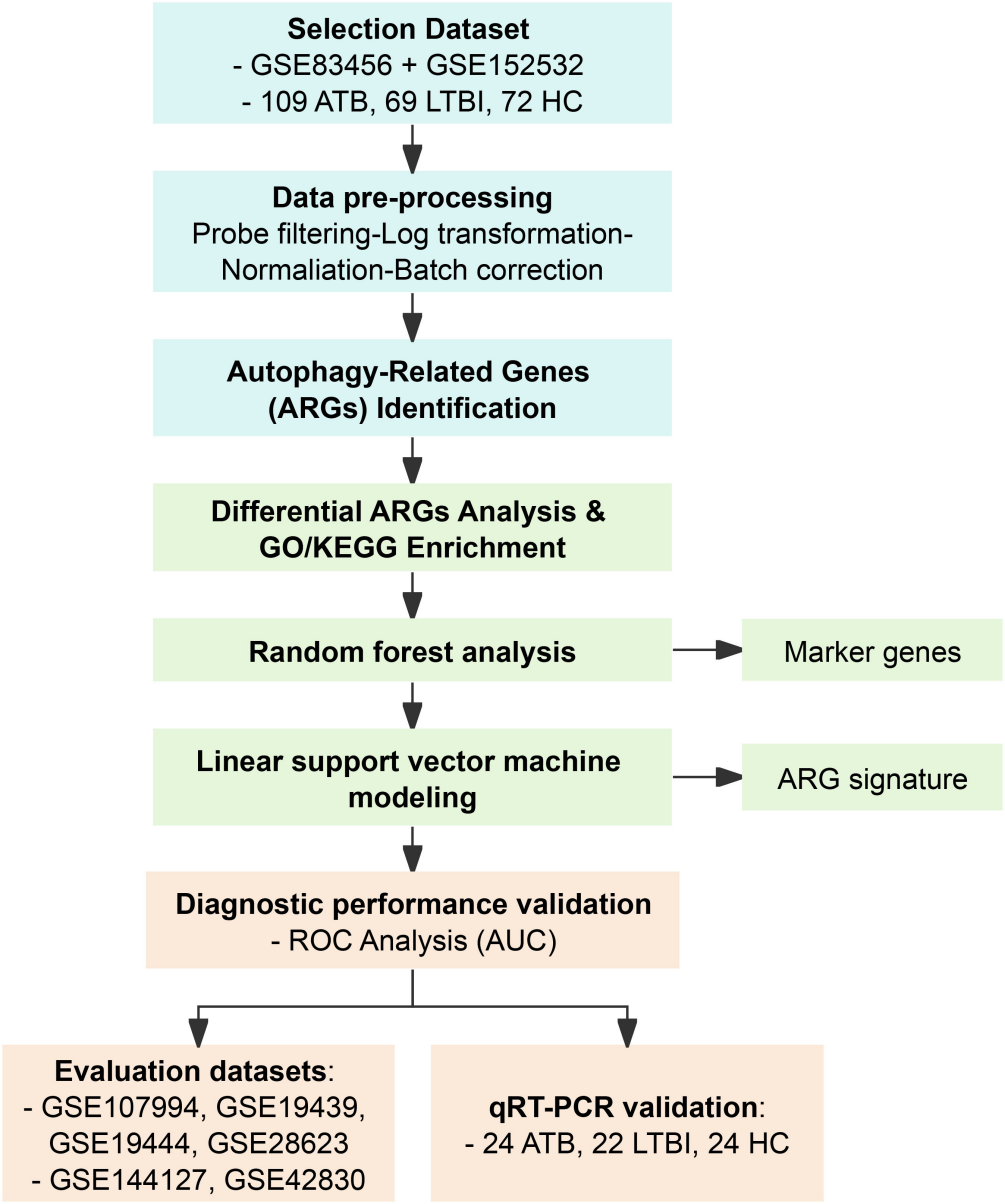
Flow chart of this study. ATB, active tuberculosis; LTBI, latent tuberculosis infection; HC, healthy control; OD, other disease.

Two microarray datasets (GSE83456 and GSE152532) generated on the Illumina Human HT-12 V4.0 Expression BeadChip were chose as the selection datasets due to platform consistency and sufficient sample size. For evaluation datasets, GSE107994, GSE19439, GSE19444, and GSE28623 were selected to represent ATB, LTBI, and HC, while GSE144127 and GSE42830 were included to evaluate specificity against OD. All datasets excluded individuals who pregnant, or participants aged <16 years. All datasets except GSE144127 excluded immunosuppressed individuals and patients receiving antimycobacterial therapy. GSE144127 contains a small subgroup of participants with comorbidities (HIV, asthma, diabetes, etc.). The characteristics of all datasets are summarized in **Table 1**, and the study design is illustrated in **Figure 1**.

**Table 1:** Summary of GEO datasets used for selection and evaluation.

Usage	Dataset	Platforms	Country	Age (mean, range, years)	Gender (Male/%)	ATB	LTBI	HC	OD	Comorbidities
Selection	GSE83456	Microarray	UK	36 (18-82)	88(58%)	92	/	61	/	Negative for HIV, diabetes, autoimmune diseases or pulmonary system diseases
	GSE152532	Microarray	UK	28 (28-60)	63(65%)	17	69	11	/	Negative for HIV and any other significant co-morbidity
Evaluation	GSE107994	High throughput sequencing	UK	38 (16-84)	103(59%)	53	72	50	/	Negative for HIV and any other significant co-morbidity
	GSE19439	Microarray	UK	32 (20-52)	24(57%)	13	17	12	/	Negative for HIV, diabetes, or autoimmune diseases
	GSE19444	Microarray	UK	37 (18-72)	31(57%)	21	21	12	/	Negative for HIV, diabetes, or autoimmune diseases
	GSE28623	Microarray	Gambia	30 (16–54)	57(53%)	46	25	37	/	Negative for HIV
	GSE144127	Microarray	UK	41 (16-87)	382(61%)	301	/	/	327	HIV 45 (ATB 25, OD 20), Asthma 54 (ATB 16, OD 38), Diabetes 54 (ATB 15, OD 39)
	GSE42830	Microarray	UK	43 (20-72)	33(58%)	16	/	/	41	Negative for HIV and any other significant co-morbidity

GEO, Gene Expression Omnibus; ATB, active tuberculosis disease; LTBI, latent tuberculosis infection; HC, healthy control; OD, other diseases.

Autophagy-related genes (ARGs) were initially compiled from two sources: the Human Autophagy Database (http://www.autophagy.lu/index.html) and the GO_AUTOPHAGY gene set available on the Gene Set Enrichment Analysis (GSEA) website (http://www.gsea-msigdb.org/gsea/msigdb). A total of 531 ARGs were identified.

### Data pre-processing

To minimize technical noise and low-abundance signals, probes with consistently low expression were systematically filtered. For each dataset (GSE83456 and GSE152532), probes were first ranked by expression intensity in all samples, then those consistently below the 50th percentile across these samples were excluded from further analysis. The remaining data was then log2-transformed, quantile normalized and merged with batch correction. Batch effect was corrected by adjusting means and standard deviations for individual probes on each dataset. As dataset GSE152532 does not consist of LTBI samples, only data from TB samples and HC samples was used to calculate means and standard deviations for correction. These two metrics were first calculated for TB class and HC class and then averaged as mean and deviation sets for each batch which were then equalized between datasets in correction. This process generated a selection dataset containing sufficient ATB, LTBI, and HC samples.

Based on the corrected probe expression level and annotations provided by the microarray manufacturer, related data were assigned to genes and the gene expression abondance was obtained by averaging the related probe expression abondance. As this study is focus on ARGs, the expression data was further filtered with the 531 ARGs mentioned previously. As a result, expression abundance for 395 ARGs were retained for next step analysis (**Supplementary Table S1**).

### Differential expression analysis

To identify the differentially expressed genes (DEGs), moderated t-statistics were applied using the “limma” R package (v4.2.0, Bioconductor 3.18) within R version 4.3.1 to compare differentially expressed autophagy-related genes (DE-ARGs) expression profiles between ATB and LTBI/HCs groups in the selection dataset. After applying the t-test, a Bonferroni correction was performed to adjust for multiple comparisons, and a significance level of 0.0001 was used to identify significant DE-ARGs. The detailed list of DE-ARGs is provided in **Supplementary Table S2**. Then, the intersection of DE-ARGs between the ATB vs LTBI comparison and ATB vs HC comparison was identified for further analysis and visualized using Venn diagrams. A heatmap of the upregulated and downregulated DE-ARGs was generated using “pheatmap” R package (v1.0.12).

### Functional enrichment analysis

Gene Ontology (GO) functional annotations were performed using the PANTHER knowledgebase (Release PANTHER18.0) (https://geneontology.org/) with a significance cutoff of 0.05. Kyoto Encyclopedia of Genes and Genomes (KEGG) pathway enrichment analysis was carried out using DAVID 6.8 under default settings (gene count ≥4 and p<0.05). The most significantly enriched GO terms and KEGG pathways were then visualized with bubble and bar plots.

### Screening marker genes with random forest

We employed Random Forest (RF)–a supervised ensemble learning method based on multiple classification trees generated from random subsets of features–to identify the most important DE-ARGs. Specifically, the ‘randomForest’ R package (v4.6.14) was used with ntree = 150. Gene importance was ranked according to the mean decrease in accuracy and the mean decrease in Gini index, and the top-ranked genes were selected as candidate marker genes.

### Evaluation of the diagnostic potential of the marker genes and signatures

To evaluate the diagnostic performance of individual marker genes, we employed logistic regression (LR) with 5-fold cross-validation using the “glmnet” R package (v4.1.8). Specifically, the selection dataset was stratified into 5 folds, ensuring each fold contained a minimum of 15 samples to maintain statistical power. In each iteration, a LR model was trained on 4 folds and tested on the remaining fold. For each fold, a Receiver Operating Characteristic (ROC) curve was generated using the “pROC” R package (v1.18.5), and its AUC was calculated. A mean ROC curve was then obtained by averaging sensitivities at corresponding specificity points across folds, and the mean AUC was reported as the primary performance metric.

For multi-gene signature construction, candidate gene combinations were identified using a linear Support Vector Machine (SVM) classifier (R package e1071 v1.7.16) with k-fold cross-validation applied to the all datasets for performance evaluation. In each cross-validation iteration, an SVM model was trained on (k-1) subgroups and validated on the remaining subgroups. ROC curves and AUC values were computed in each iteration, followed by calculation of a mean ROC curve and mean AUC after all iterations. The optimal gene combination was selected based on the highest cross-validation performance within the selection dataset. To maintain the generalizability of our findings, all SVM parameters except for the fold number of cross-validation were deliberately kept identical across all datasets. The k-fold values was adjusted with dataset size and variability control: k=3 for GSE19439, GSE19444 and GSE42830; k=5 for the the selection dataset, GSE107994, GSE28623 and GSE144127. Model performance was evaluated on the evaluation datasets to assess the signature’s ability to differentiate: ATB vs. LTBI, ATB vs. HC, and, where applicable, ATB vs. other diseases (OD).

### Study participants and sample collection

A total of 70 participants including 24 ATB patients, 22 LTBI individuals, and 24 HCs were recruited from Jiangxi Provincial Chest Hospital, Jiangxi, China. ATB samples were collected from active pulmonary tuberculosis patients which confirmed by at least one positive Mtb culture or nucleic acid amplification test of sputum samples. LTBI patients were defined by positive T-SPOT.TB results, no clinical symptoms of tuberculosis, no history of tuberculosis, and normal chest X-ray. HCs were defined by negative T-SPOT.TB results, no tuberculosis-related symptoms, no history of tuberculosis, and normal chest X-ray. All participants were aged 18–50 years. Individuals with HIV infection, autoimmune illnesses, current immunosuppressive therapies, pregnancy, non-TB pulmonary diseases, or recent antimycobacterial treatment (within 6 months) were excluded. Peripheral blood of 3 mL from each participant was collected in heparinized Vacutainer tubes and stored at −80°C until analysis. The study was approved by the Ethics Committee of Jiangxi Provincial Chest Hospital, and all participants provided written informed consent.

### RNA isolation and reverse transcription quantitative PCR analysis

The mRNA expression of candidate marker genes were analyzed by Reverse Transcription quantitative PCR (RT-qPCR). Total RNA was extracted using TRIzol reagent (Invitrogen, MA, USA), according to the manufacturer’s instructions. Five microliters of RNA were used in the One Step RT-qPCR Probe Kit (Q231, Vazyme, Jiangsu, China), and RT-qPCR was carried out on a SLAN-96S Real-time PCR System (Hongshi, Shanghai, China). β-actin served as reference gene, and relative gene expression levels were calculated using the 2^(-ΔΔCT) method. Primer and TaqMan probe sequences for all target genes are listed in the **Supplementary Table S3**. Subsequently, expression data were analyzed by one-way analysis of variance (ANOVA) followed by Tukey’s *post hoc* test for multiple group comparisons, using GraphPad Prism 9.0.0 (GraphPad, USA), and a p-value < 0.05 was considered statistically significant. Finally, to further assess the diagnostic utility of the ARG signature, we built a SVM model with 5-fold cross-validation and used ROC curves to measure the ability of the signature to differentiate ATB from LTBI or HC. The AUC was then reported as the principal performance metric.

## Results

### DE-ARGs identification in ATB patients

**Figure 2:**
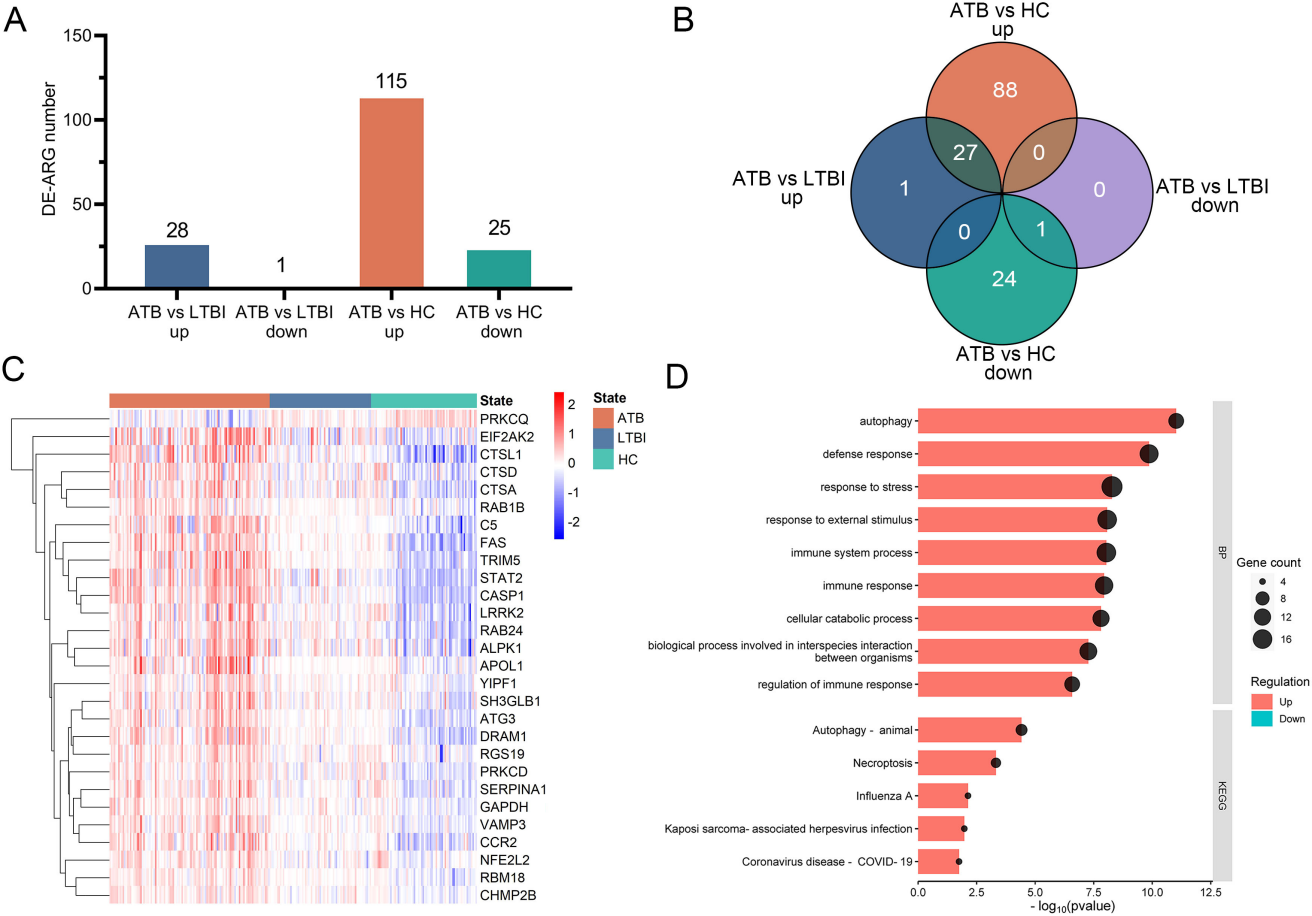
Identification of DE-ARGs among ATB, LTBI, and HC in the selection datasets. **(A)** The numbers of significantly up-regulated or down-regulated DE-ARGs in the ATB vs. LTBI and ATB vs. HC comparisons. **(B)** Venn diagrams illustrating the intersection number of DE-ARGs between the two comparisons. **(C)** Heatmap of relative gene expression levels of the 28 overlapping DE-ARGs across ATB, LTBI, and HC groups. Gene expression were scaled using (x-mean)/mean to show relative expression above (red) or below (blue) the gene’s mean. **(D)** Bubble and bar plots display the top enriched GO Biological Process terms (upper panel) and KEGG pathways (lower panel) for these DE-ARGs; for GO analysis, the top nine terms were selected based on p-value ranking, while KEGG pathways with an overlap of more than five genes were included. DE-ARG, differentially expressed autophagy-related gene; ATB, active tuberculosis; LTBI, latent tuberculosis infection; HC, healthy control.

Two microarray datasets (GSE83456 and GSE152532) were merged to create a selection dataset containing 109 ATB, 69 LTBI, and 72 HC samples. Focusing on differentially expressed autophagy-related genes (DE-ARGs), we identified 140 DE-ARGs in ATB vs. HC (115 upregulated and 25 downregulated) and 29 DE-ARGs in ATB vs. LTBI (28 upregulated and 1 downregulated) (**Figure 2A**). As illustrated by the Venn diagram (**Figure 2B**), 28 genes (27 upregulated, 1 downregulated) were identified across ATB vs. HCs and ATB vs. LTBI. A heatmap of these 28 genes showed distinct clustering that clearly separates the ATB group from both the LTBI and HC groups (**Figure 2C**).

### Functional enrichment analysis of DE-ARGs

The functions of the 28 identified DE-ARGs were explored through GO and KEGG pathway enrichment analyses. GO analysis showed significant enrichment of DE-ARGs in Biological Process (BP) terms such as ‘autophagy’, ‘defense response’, ‘response to stress’, ‘response to external stimulus’, ‘immune system process’ and ‘immune response’ (**Figure 2D**, upper panel). Furthermore, the KEGG pathway enrichment analysis revealed significant associations of these DE-ARGs with pathways including ‘autophagy-animal’, ‘necroptosis’, and ‘influenza’ (**Figure 2D**, lower panel). These findings underscored the connection of the 28 DE-ARGs and immune regulation.

### Identification of marker ARGs via random forest classifier

**Figure 3:**
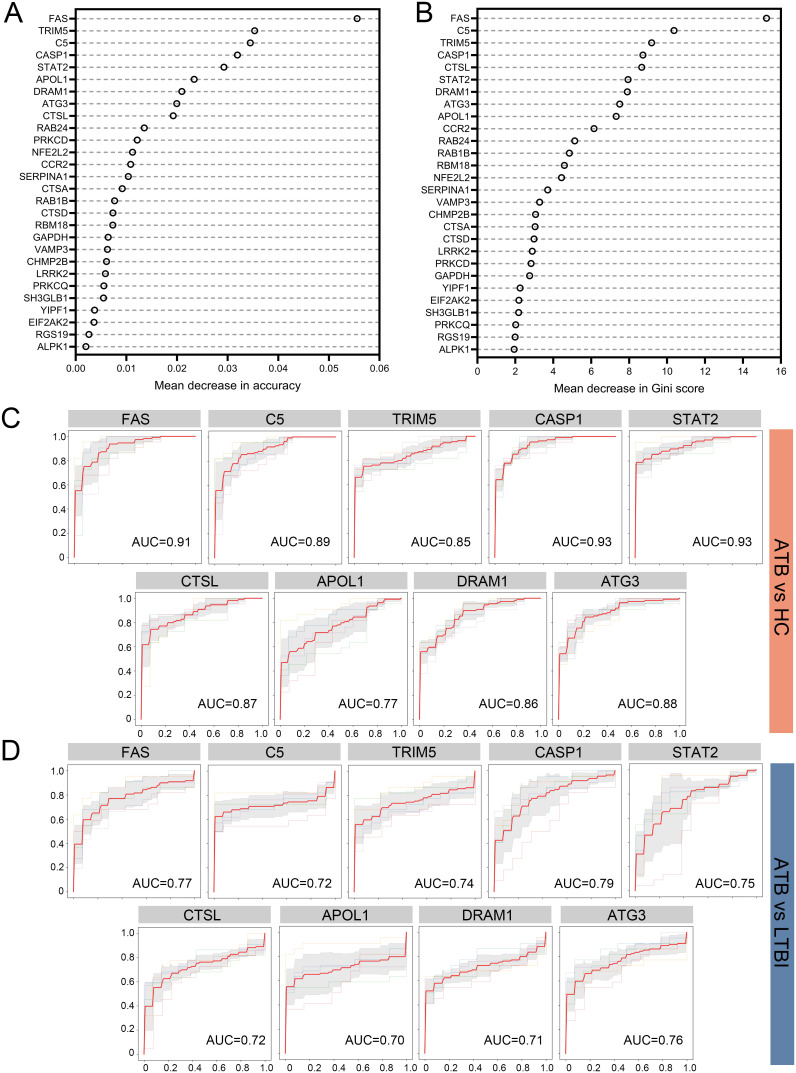
Screening of marker ARGs for ATB using a Random Forest classifier and diagnostic performance evaluation in the selection dataset. **(A)** Mean decrease in accuracy for each DE-ARG in the Random Forest model. **(B)** Mean decrease in Gini index for each DE-ARG in the Random Forest model. ROC curves for distinguishing ATB from HC **(C)** and ATB from LTBI **(D)** using individual DE-ARGs. Sensitivity is plotted against 1-Specificity. Thin lines represent the ROC curves for each fold; bold red lines indicate the mean ROC curve. Shaded regions represent standard deviation across folds. Mean AUC values derived from 5-fold cross-validation are displayed for each comparison. DE-ARG, differentially expressed autophagy-related gene; ATB, active tuberculosis; LTBI, latent tuberculosis infection; HC, healthy control; ROC, receiver operating characteristic; AUC, area under the ROC curve.

To select reliable diagnostic markers for ATB, we constructed a Random Forest (RF) model in the selection dataset and assessed the importance of the 28 DE-ARGs according to mean decrease in accuracy (**Figure 3A**) and mean decrease in Gini (**Figure 3B**). Nine genes were consistently ranked in the top 10 by both metrics, namely *FAS*, *C5*, *TRIM5*, *CASP1*, *CTSL*, *STAT2*, *DRAM1*, *ATG3*, and *APOL1*.

We next evaluated the ability of each gene to distinguish ATB from HC and ATB from LTBI in the selection dataset. As shown in **Figure 3C** (ATB vs. HC), three genes (*CASP1*, *STAT2* and *FAS*) achieved AUC values above 0.9, while *C5*, *ATG3*, *CTSL*, *DRAM1*, and *TRIM5* showed moderate performance (AUC 0.8-0.89) and APOL1 had a lower AUC of 0.77. For ATB vs. LTBI (**Figure 3D**), *CASP1* again showed the highest AUC (0.79), whereas most of the remaining genes ranged from 0.70 to 0.79, with *APOL1* showing the lowest AUC of 0.70. In diagnostic accuracy studies, the AUC values are generally interpreted as follows: 0.6-0.7 indicates poor discriminatory capacity, 0.7-0.8 indicates fair diagnostic performance, 0.8-0.9 indicates considerable accuracy, and values above 0.9 indicates excellent classification ability (Nahm, 2022). Given that *APOL1* exhibited AUC values below 0.8 in both the ATB vs. HC and ATB vs. LTBI comparisons, this gene was excluded from subsequent analysis. Based on these findings, *FAS*, *C5*, *TRIM5*, *CASP1*, *CTSL*, *STAT2*, *DRAM1*, and *ATG3* were collectively regarded as marker ARGs, with APOL1 excluded due to relatively lower performance.

### Construction and evaluation of the ARG signatures in ATB diagnosis

**Figure 4:**
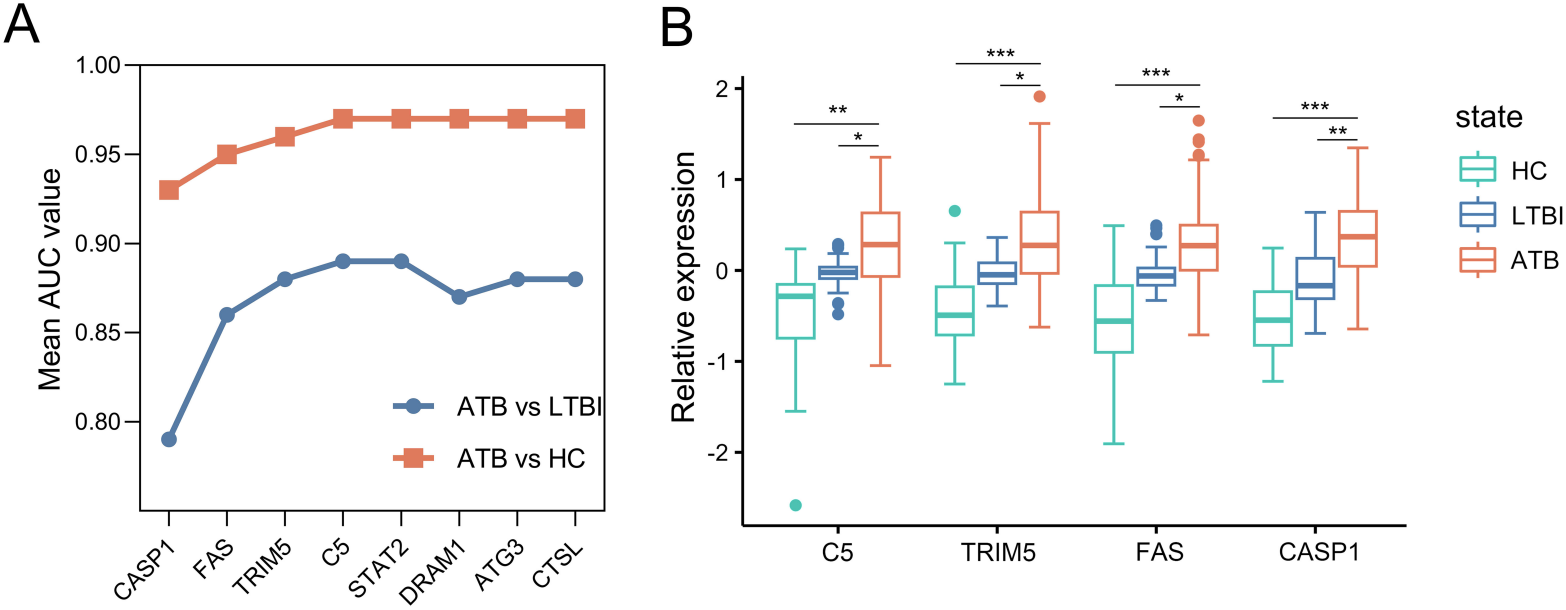
Construction of the ARG signature for identifying ATB in the selection dataset. **(A)** Iterative performance of the greedy forward feature selection algorithm for signature construction. Mean AUC values (y-axis) derived from 5-fold cross-validation are shown for distinguishing ATB from HC (orange) and ATB from LTBI (blue) as genes are sequentially added to the signature (x-axis). **(B)** Box plots depicting relative expression of the four selected ARGs across ATB, LTBI, and HC groups. Each gene expression abondance was scaled using (x-mean)/mean to show relative expression. Boxplots were then generated with ggplot2 (v3.4.2). Boxes span the 25th-75th percentiles (interquartile range, IQR), with the median line; whiskers extend to 1.5×IQR; individual points represent outliers beyond the whiskers. Statistical significance was calculated by a moderated t test. *p value < 10^-2^; **p value <10^-8^; ***p value < 10^-18^. ARG, autophagy-related gene; ATB, active tuberculosis; LTBI, latent tuberculosis infection; HC, healthy control; ROC, receiver operating characteristic; AUC, area under the ROC curve.

Then, we constructed ARG signatures from the eight marker ARGs identified above. The evaluation of these signatures began with *CASP1*, the gene with the highest individual performance in the selection dataset. Additional marker ARGs were sequentially added, each contributing the largest increase in the signature’s AUC value at every step. As shown in **Figure 4A**, the combination of *CASP1*, *FAS*, *TRIM5*, and *C5* resulted in an AUC of 0.97 for distinguishing ATB from HC, with no further improvement observed by adding additional genes. For distinguishing ATB from LTBI, the combination of *CASP1*, *FAS*, *TRIM5*, and *C5* again achieved the highest AUC of 0.89, while the inclusion of *DRAM1*, *ATG3*, and *CTSL* led to a decrease in the model’s discriminatory ability. Based on these results, *CASP1*, *FAS*, *TRIM5*, and *C5* were selected to construct a four-gene signature. Furthermore, microarray data from the selection dataset demonstrated a significant increase in mRNA expression levels of *CASP1*, *FAS*, *TRIM5*, and *C5* in the ATB group compared to both LTBI and HC groups, supporting their utility as diagnostic markers for ATB (**Figure 4B**).

### Evaluation of the ARG signature for predicting ATB versus LTBI/HC in independent datasets

**Figure 5:**
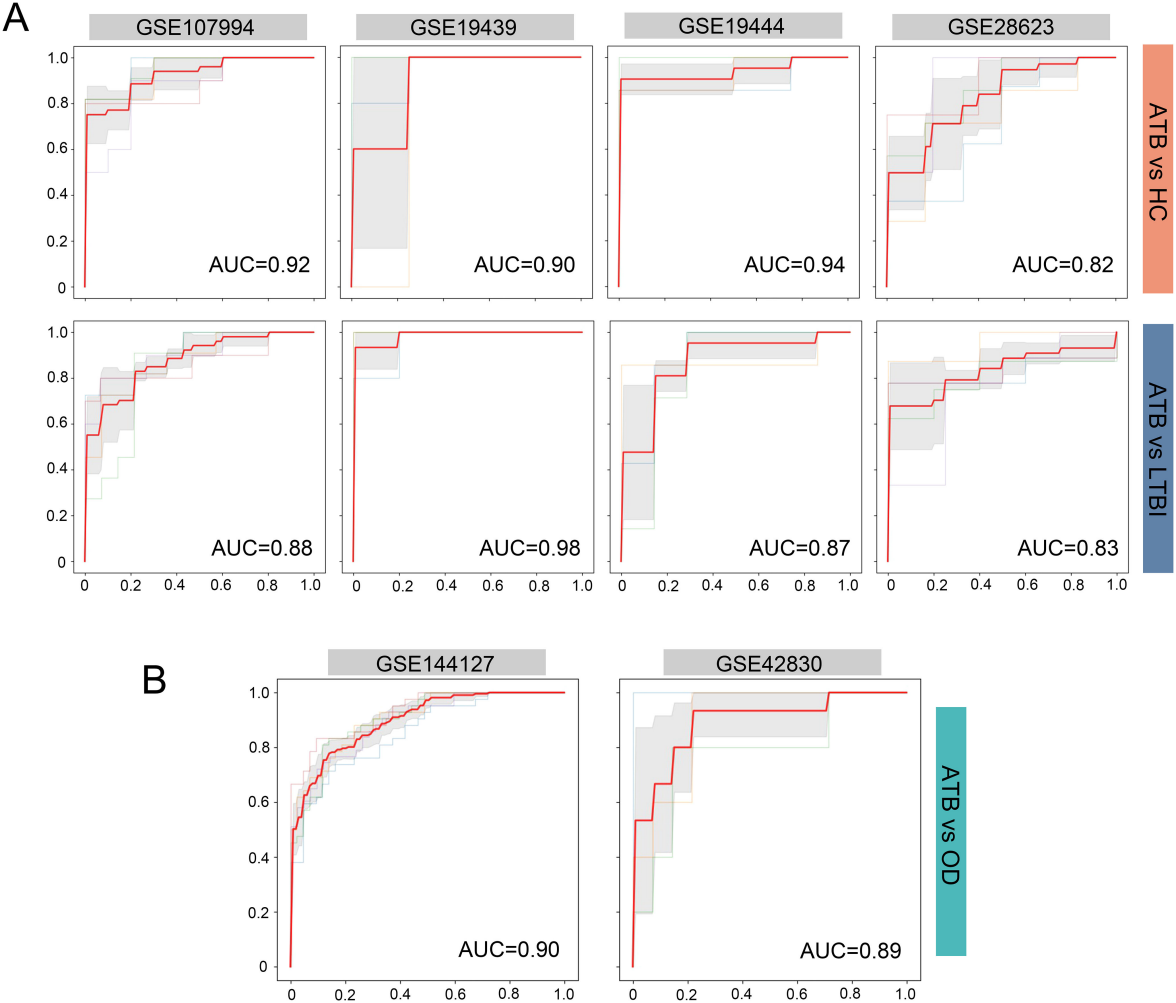
Evaluation for the four-ARG signature in independent datasets. **(A)** ROC curves for distinguishing ATB from HC and ATB from LTBI in the datasets GSE107994, GSE19439, GSE19444, and GSE28623. **(B)** ROC curves for distinguishing ATB from OD in the GSE144127 and GSE42830 datasets. Sensitivity is plotted against 1-Specificity. Thin lines represent the ROC curves for each fold; bold red lines indicate the mean ROC curve. Shaded regions represent standard deviation across folds. Mean AUC values derived from cross-validation are displayed for each comparison. ARG, autophagy-related gene; ROC, receiver operating characteristic; ATB, active tuberculosis; LTBI, latent tuberculosis infection; HC, healthy control; OD, other diseases; AUC, area under the ROC curve.

We evaluated the diagnostic performance of the four-gene ARG signature in distinguishing ATB from LTBI and HC using four independent evaluation datasets (GSE107994, GSE19439, GSE19444, and GSE28623). ROC curve analysis was performed for each dataset, as shown in **Figure 5A**. The four-gene signature demonstrated AUC values ranging from 0.82 to 0.94 for distinguishing ATB from HC, and from 0.83 to 0.98 for distinguishing ATB from LTBI. These results indicate that the four-gene ARG signature provides excellent diagnostic accuracy across different independent datasets.

### Prediction accuracy of the ARG signature in distinguishing ATB from other diseases

We assessed the predictive accuracy of the four-gene ARG signature for distinguishing individuals with ATB from those with other diseases (OD) that clinically resemble tuberculosis. This evaluation was conducted using two independent datasets (GSE144127 and GSE42830). As shown in **Figure 5B**, the ARG signature achieved AUC values of 0.90 and 0.89 in the GSE144127 and GSE42830 datasets, respectively, demonstrating its strong discriminatory capability, even in the challenging task of differentiating ATB from other similar clinical presentations.

### RT-qPCR validation

**Figure 6:**
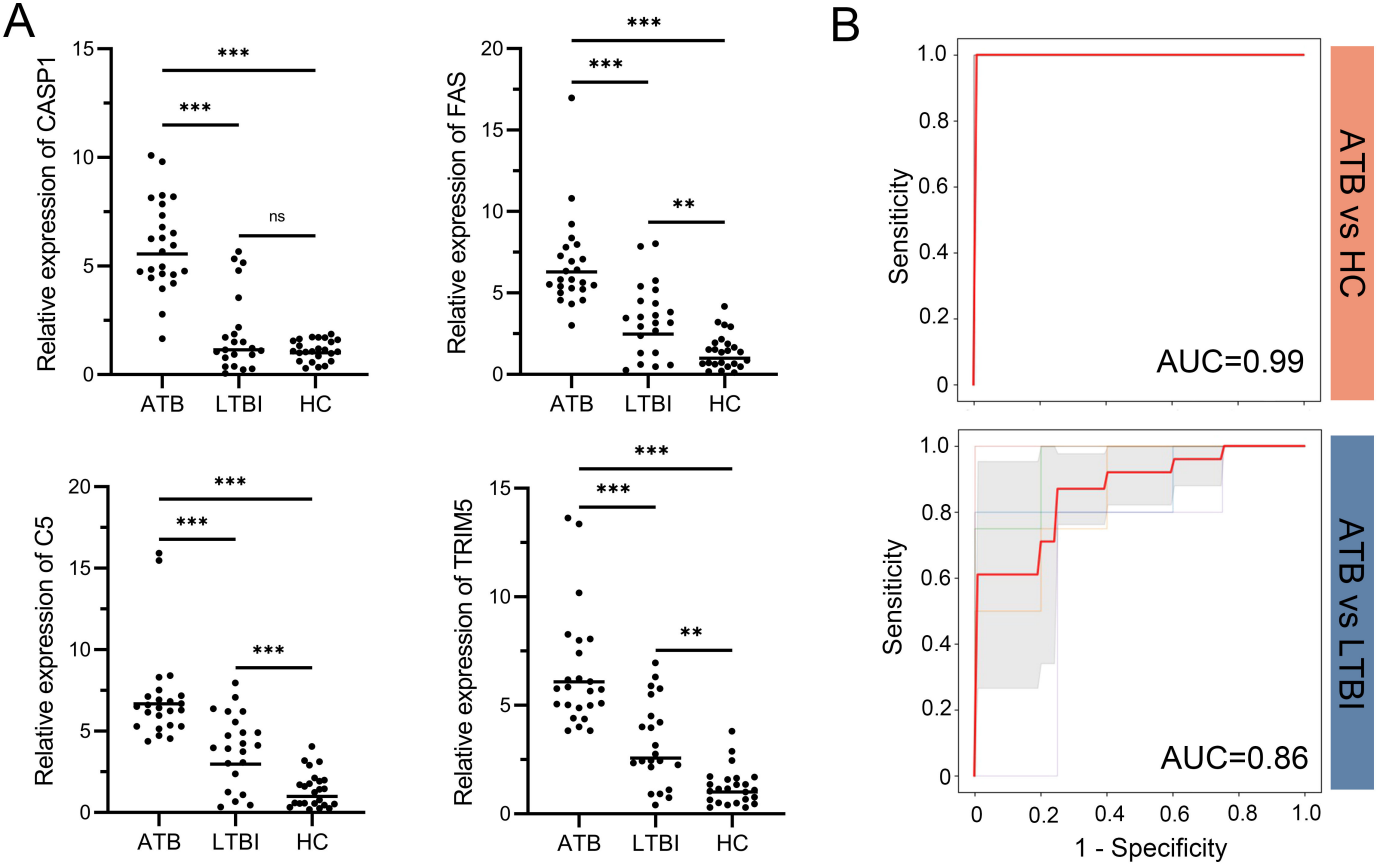
RT-qPCR validation of the four-gene signature and its diagnostic performance. **(A)** Relative mRNA expression of *CASP1*, *FAS*, *TRIM5*, and *C5* in whole blood samples from ATB, LTBI, and HC groups. Expression values were normalized to reference genes and transformed fold changes relative to the HC group. Points represent individual samples; horizontal lines indicate group medians. Statistical significance was calculated by one-way ANOVA followed by Tukey’s *post hoc* test. **p < 0.01; ***p < 0.001; ns, not significant. **(B)** ROC curves of the four-gene signature in distinguishing ATB from HC (top) and ATB from LTBI (bottom). Thin lines represent the ROC curves for each fold; bold red lines indicate the mean ROC curve. Shaded regions represent standard deviation across folds. Mean AUC values derived from 5-fold cross-validation are displayed for each comparison. ATB, active tuberculosis; LTBI, latent tuberculosis infection; HC, healthy control; ROC, receiver operating characteristic; AUC, area under the ROC curve.

The mRNA expression levels of *CASP1*, *FAS*, *TRIM5*, and *C5* were validated in whole blood samples from 24 ATB patients, 22 LTBI patients, and 24 HC participants. Statistical analysis indicated that these genes were significantly more highly expressed in the ATB group compared to both the LTBI and HC groups (**Figure 6A**). Furthermore, a SVM model with 5-fold cross-validation was constructed to evaluate the discriminative ability of the four-gene signature. ROC curve analysis showed that the four-gene signature achieved excellent differentiation between ATB and HC, with an AUC value of 0.99, and also demonstrated good performance in distinguishing ATB from LTBI, with an AUC value of 0.86 (**Figure 6B**).

## Discussion

The clinical progression of Mtb infection hinges on the dynamic interplay between bacterial immune evasion and host defense mechanisms (Kaufmann et al., 2018; Chai et al., 2020). Our study identified a novel four-gene autophagy-related signature (*CASP1*, *FAS*, *TRIM5*, and *C5*) that robustly discriminated ATB from LTBI, HCs, and OD. The signature demonstrated high diagnostic accuracy across multiple independent cohorts (AUC 0.82-0.98) and was validated by RT-qPCR in clinical samples (AUC 0.86-0.99). Notably, these genes are functionally linked to autophagy and immune regulation, aligning with the critical role of host-pathogen interactions in TB progression. These findings highlight the translational potential of targeting autophagy-associated genes for ATB diagnostics.

The role of autophagy in Mtb infection has evolved over two decades from a canonical degradative pathway to a multifaceted host-pathogen interface (Gutierrez et al., 2004). Canonical autophagy, mediated by ATG proteins such as ATG5, restricts Mtb survival in alveolar macrophages by promoting phagosome-lysosome fusion and suppressing neutrophil-driven inflammation during early infection (Kimmey et al., 2015; Kinsella et al., 2023). Recent studies has demonstrated that complete loss of core autophagy components (e.g., ATG7, ATG16L1) increased host susceptibility by allowing pathogen-induced phagosome damage and macrophage necrosis (Aylan et al., 2023; Golovkine et al., 2023). ATG5 deficiency not only disrupts bacterial containment through impaired autophagy but also causes pathological neutrophil activation due to defective lysosomal repair (Wang et al., 2023). This mechanistic duality is mirrored in Mtb’s evolved counterstrategies. Virulent strains subvert autophagy through host autophagy pathways through their virulence effector proteins to promote intracellular survival. Exemplary evasion mechanisms include ESX-1 type VII secretion system-induced phagosomal membrane rupture in macrophages (Wong, 2017), PDIM-mediated blockade of LC3-associated phagocytosis (Mittal et al., 2024), and SapM/Eis-dependent inhibition of autophagosome maturation (Duan et al., 2016). The dual nature of autophagy–balancing bacterial control and immunopatholo–positions it as a compelling biomarker candidate, reflecting both pathogen burden and host immune status (Kimmey and Stallings, 2016).

Our autophagy-derived four-gene signature (*CASP1*, *FAS*, *TRIM5*, *C5*), identified through functional screening of autophagy regulators in Mtb infection, bridges canonical degradative pathways with broader immune networks. Functional enrichment analysis of the 28 DE-ARGs revealed their strong association with immune pathways (**Figure 2**), providing a mechanistic foundation for their multifaceted roles. TRIM5, an E3 ubiquitin ligase, bridges selective autophagy and innate immunity by enhancing its interaction with ULK1/Beclin-1 complexes (Mandell et al., 2014). An increase in TIRM5 was observed in Mtb-infected human monocyte-derived macrophages (Romagnoli et al., 2023). CASP1 is a critical enzyme that links autophagy to inflammasome signaling pathways and pyroptosis (Boucher et al., 2018; Lu et al., 2020). Overexpression of CASP1 have been shown to restrict Mtb replication in macrophages (Mishra et al., 2010). FAS, a classic apoptosis modulator activated by Fas ligand (FasL) engagement, reduces intracellular Mtb replication in macrophages (Li et al., 1998; Mustafa et al., 2005). C5 is a component of the complement system and essential for host defense against Mtb, as C5-deficient mice exhibit increased susceptibility to infection (Actor et al., 2001). Furthermore, C5 deficiency in T cells impairs IFNγ production upon Mtb stimulation (Mashruwala et al., 2011). Collectively, these genes exemplify autophagy’s role as a signaling hub, intersecting with inflammasome signaling, apoptosis, and complement activation to provide a multi-dimensional biomarker signature for ATB detection.

Extensive transcriptomic studies have identified blood-based signatures for tuberculosis diagnosis, yet clinical implementation barriers persist. A systematic comparison of 16 signatures showed higher numbers of gene in signature would not increase the accuracy of the signature in cross-sectional cohorts (Warsinske et al., 2019). Moreover, signatures with larger gene number, such as Berry393 (Berry et al., 2010) or Anderson51 (Anderson et al., 2014), may pose technical and economic challenges for PCR-based assay development. Recent advancements prioritized RT-qPCR-compatible signatures, exemplified by the Sweeney3 panel (commercialized as Xpert MTB HR), which meets WHO TPP benchmarks for distinguishing ATB from HCs (Mendelsohn et al., 2022). Through machine learning-driven selection of autophagy-related pathways, we derived a four-gene signature (*CASP1*, *FAS*, *TRIM5*, *C5*) addressing two diagnostic challenges. First, it targets the critical need for ATB-LTBI discrimination, arising from shared Mtb-specific immune responses. Previous study reported that reduced accuracy in ATB vs. LTBI (AUC 0.739) than ATB vs. HCs (AUC 0.892) (Wu et al., 2023). The AUC of our signature for ATB vs. LTBI reached 0.83-0.98 across four evaluation cohorts and 0.86 in clinical specimens, suggesting the four genes enhance immunological specificity. Second, the signature demonstrates robustness against TB-mimicking diseases. The difficulty of differentiating ATB from ODs has been exemplified by two recent prospective evaluations (Turner et al., 2020; Hoang et al., 2021). We validated our signature against two datasets (GSE144127 and GSE42830). Participants in GSE144127 were recruited during routine practice, and the OD group comprised a diverse conditions (pneumonia, sarcoidosis, cancer, non-pneumonia lower respiratory tract infection, bronchiectasis, asthma, or atypical *Mycobacterium* spp. infection). While the Sweeney3 signature achieved AUC 0.83 in distinguishing all active TB from other disease in GSE144127 (Hoang et al., 2021), our four-gene panel demonstrated enhanced diagnostic power (AUC 0.90 for ATB vs ODs). The Fujifilm SILVAMP TB LAM (FujiLAM) is a next-generation point-of-care urine-based assay designed to identify tuberculosis with high specificity (Broger et al., 2019). A recent diagnostic test accuracy study reported a sensitivity of 53.2% and specificity of 98.9% in differentiating HIV-negative TB patient from not-tuberculosis participants (Broger et al., 2020). In comparison, when applying the same specificity threshold of 98.9% to our clinical cohort data, our signature demonstrated a sensitivity of 84.1% in distinguishing ATB from HCs – higher than that of FujiLAM. Although both tests are cost-efficient, FujiLAM’s reported cost of US$6 per test exceeds that of Xpert HR (US$2 per test, PCR-based test) (Reddy et al., 2021; Gupta-Wright et al., 2024). This suggests our assay could achieving better performance with lower per-test cost than FujiLAM. Altogether, the combination of biological relevance and technical feasibility positions our signature as a pragmatic solution for WHO TPP-aligned TB diagnostics.

Despite these advances, our study has several limitations. First, the limited sample size in evaluation datasets reduced statistical power. To enhance reliability and clinical applicability, future validation across multiple public datasets and larger prospective cohorts is needed. Second, the exclusion of children (<16 years), individuals living with HIV, patients with comorbidities (e.g., diabetes), and subclinical TB patients, restricts the clinical generalizability of our findings. Prospective cohorts incorporating these underrepresented populations are needed to improve the signature’s applicability. Third, evaluation datasets predominantly comprised participants from the UK (low-TB-incidence, high-resource setting), which may not reflect diagnostic challenges in endemic regions. The signature could be tested in cohorts from other TB endemic regions. Fourth, reliance on self-reported TB exposure history or prior TB episodes in some cohorts may introduce recall bias, potentially affecting patient inclusion/exclusion criteria. Finally, technical discrepancies between transcriptomic platforms and PCR-based assays pose reproducibility risks. Although we validated the signature in a small clinical cohort using RT-qPCR, platform-specific biases remain unresolved. Prior to large-scale prospective trials, standardization and cross-platform calibration of RT-qPCR assays are essential to ensure signature reproducibility across diverse laboratory environments.

In conclusion, we identified a novel autophagy-related four-gene signature (*CASP1*, *FAS*, *TRIM5*, *C5*) that robustly discriminated ATB from LTBI, HCs, and ODs across selection, evaluation, and clinical blood cohorts. The signature achieved high diagnostic accuracy for ATB-LTBI differentiation and maintaining robustness against diverse TB-mimicking diseases. These findings provide strong evidence for the establishment of a blood-based ATB diagnosis strategy, with potential to enhance ATB detection accuracy and reduce TB transmission risks through prompt treatment initiation.

## Data Availability

Publicly available datasets were analyzed in this study. This data can be found here: NCBI: GSE83456, GSE152532, GSE107994, GSE19439, GSE19444, GSE28623, GSE144127 and GSE42830.
